# AMENet is a monocular depth estimation network designed for automatic stereoscopic display

**DOI:** 10.1038/s41598-024-56095-1

**Published:** 2024-03-11

**Authors:** Tianzhao Wu, Zhongyi Xia, Man Zhou, Ling Bing Kong, Zengyuan Chen

**Affiliations:** 1 College of New Materials and New Energies, Shenzhen University of Technology, Shenzhen, 518118 Guangdong China; 2 College of Applied Technology, Shenzhen University, Shenzhen, 518060 Guangdong China

**Keywords:** Depth loss, Monocular depth estimation, CNN, Transformer, Computer science, Displays

## Abstract

Monocular depth estimation has a wide range of applications in the field of autostereoscopic displays, while accuracy and robustness in complex scenes are still a challenge. In this paper, we propose a depth estimation network for autostereoscopic displays, which aims at improving the accuracy of monocular depth estimation by fusing Vision Transformer (ViT) and Convolutional Neural Network (CNN). Our approach feeds the input image as a sequence of visual features into the ViT module and utilizes its global perception capability to extract high-level semantic features of the image. The relationship between the losses is quantified by adding a weight correction module to improve robustness of the model. Experimental evaluation results on several public datasets show that AMENet exhibits higher accuracy and robustness than existing methods in different scenarios and complex conditions. In addition, a detailed experimental analysis was conducted to verify the effectiveness and stability of our method. The accuracy improvement on the KITTI dataset compared to the baseline method is 4.4%. In summary, AMENet is a promising depth estimation method with sufficient high robustness and accuracy for monocular depth estimation tasks.

## Introduction

Acquiring depth information is a crucial task for machines to perceive the objective reality of a scene from 2D images^1^. Depth estimation can be achieved by utilizing two input images of the same scene captured from distinct viewpoints^2^, a technique referred to as binocular depth estimation. Recent studies have demonstrated that humans in the real world rely on images obtained from their eyes to estimate the depth of surrounding objects. Thus, depth estimation stands as a classical task in the realm of computer vision, finding wide-ranging applications in domains, such as object tracking and autonomous driving^3–5^. From cost perspective, high-quality monocular depth estimation holds appeal, as it can substitute for laser radar sensors, thus offering greater flexibility and affordability. Traditional approaches often involve manual crafting^6^ and rely on visual cues (shadows, textures, etc.) or employ supplementary information^7^. For multi-view auto-stereoscopic displays, the conventional approach involves capturing the same scene from multiple viewpoints at varying angles to obtain left and right images with depth information^8,9^. Evidently, this dependency on additional sensors diverges from the original intent of monocular depth estimation. In depth estimation tasks, providing the depth value corresponding to each pixel is essential. Within dense prediction tasks, depth learning-based methods primarily fall into two categories. One is based on image patch tasks, utilizing small neighborhoods around pixels or superpixel blocks for independent classification (using fully connected layers, hence requiring fixed image patch sizes). The other relies on fully convolutional networks for pixel-to-pixel prediction, enabling segmentation of images of arbitrary sizes without the need for classifying each image patch.

**Figure 1:**
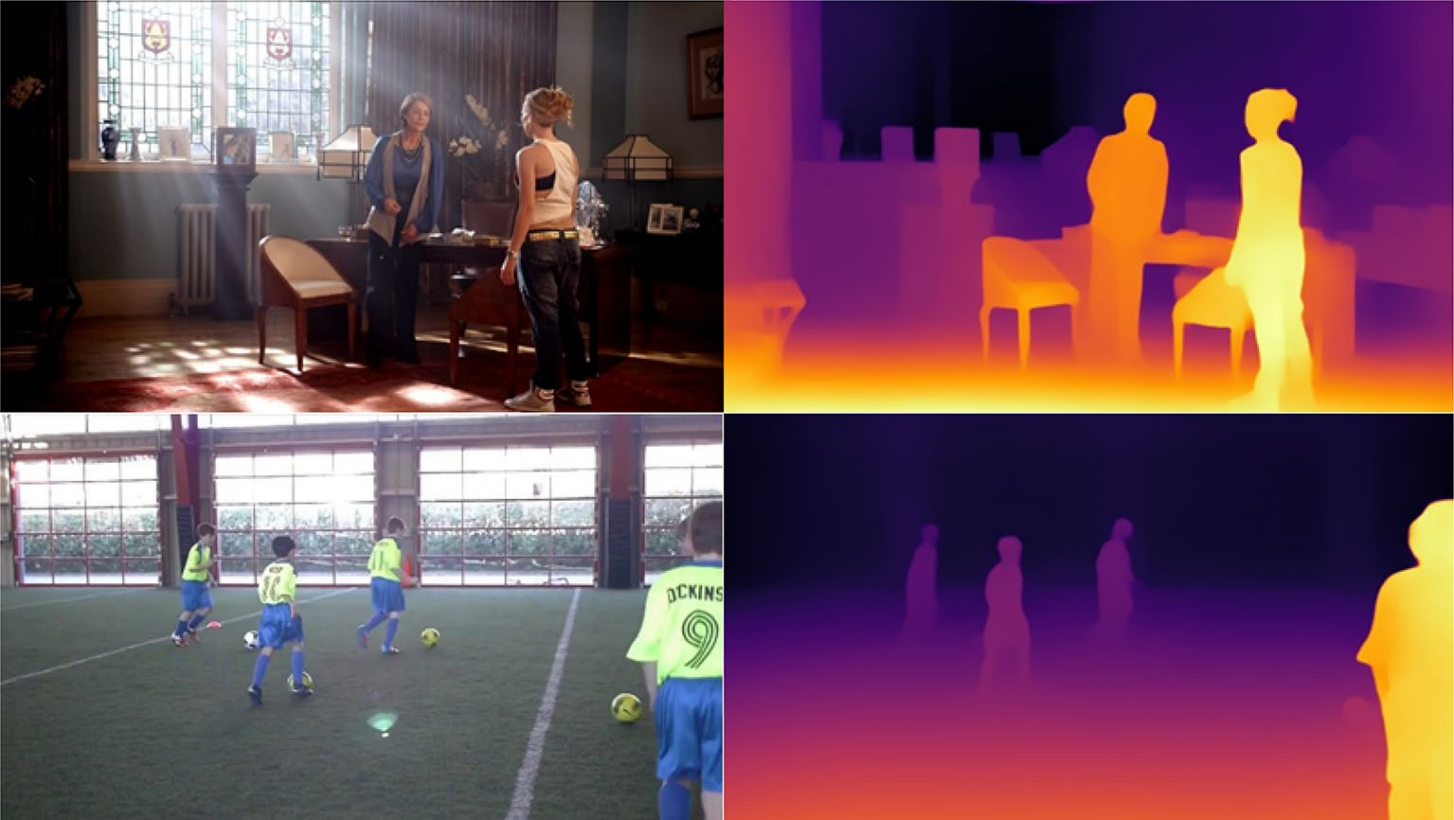
AMENet's predictions for indoor and outdoor scenes.

With the advancement of deep learning techniques, training CNN using well-designed loss functions and extensive annotated datasets has shown effectiveness in predicting depth maps from single images^10^. However, in practical applications, the actual performance of monocular depth estimation often falls short of expectations. It demands substantial datasets for training, making it challenging for real-time auto-stereoscopic display devices. To address this concern, we explore existing deep learning models, specifically those based on CNN and ViT^11^. We propose a novel architecture based on Vision Transformers to tackle this task, with modifications leading to the development of AMENet model. Throughout our training process, we introduce a segmentation lens and leverage a custom dataset for a novel multi-task learning approach. Typical monocular depth estimation algorithms can be roughly categorized into three groups: supervised algorithms, unsupervised algorithms, and video-based depth estimation methods. Supervised algorithms address known problems, training models using labeled data to perform specific tasks and predicting known outcomes from input two-dimensional images to output depth maps. Given the difficulty in obtaining depth data, many algorithms resort to unsupervised models that jointly train on binocular image data captured by using two cameras. These binocular images can predict each other, thereby obtaining corresponding disparity data, which can then be translated into depth information based on the disparity-depth relationship. Alternatively, the correspondence problem between pixels in binocular images is treated as a stereo matching task for training. The third category involves video-based depth estimation, encompassing both single-frame monocular depth estimation and pixel-wise stereo matching in multi-frame videos to acquire multi-view images and estimate camera poses. Due to the need for labeled training material, adjusting weights, and quantifying depth map losses, we will employ a "supervised training" approach. Our network is based on CNN and ViT. The choice of models does not require downloading the original ones, referencing them to be sufficient. We will provide a qualitative comparison against alternative methods. Figure 1 shows the predictions of our model.

## Related works

CNN have found widespread applications in computer vision^12,13^. The layout of convolutional operations significantly enhances the effectiveness of neural networks by incorporating contextual information, weight sharing, and translation invariance. CNN have become a predominant approach in the research field of intelligent visual systems. However, many CNN employ 3 × 3 convolutions, which limit the network's receptive field^14^. In dense prediction tasks, such as semantic segmentation, object detection, and depth estimation, a larger receptive field is crucial for establishing contextual consistency. In the case of monocular depth estimation, global contextual information can smooth the disparities in input feature maps, resulting in accurate depth information. Presently, most approaches expand the receptive field of convolutions by stacking multiple convolutional layers^15^. For downstream tasks, CNN backbone networks with extensive receptive fields are also gradually emerging^16^. Within stacked network architectures, the encoder-decoder configuration is the most commonly employed for monocular depth estimation tasks.

Transformers were originally designed to capture long-range correlations in textual information, which is why they quickly found applications in the field of computer vision^17^. The self-attention mechanism employed in transformers is a special form of attention, which works effectively in capturing distant dependencies between two pixels. As a result, transformers are playing an increasingly important role in the realm of visual tasks. For certain visual tasks, various self-attention networks demonstrate superior performance over mainstream CNN. For instance, in the case of DETR, transformers are used for dense prediction, dividing the input image into multiple patches that are then merged^18^. Solely relying on self-attention mechanisms could cause the network to overlook correlations between feature map channels, while this globally designed pattern could struggle with detecting small objects. Building upon this, LocalViT introduces locality to the vision transformer by incorporating deep convolutions in the feedforward network^19^. However, due to the addition of extra modules, the inference speed is consequently reduced. The emergence of ViT allows us to treat image data similarly to natural language processing, yet ViT does not fully leverage the spatial structural information within images. Solely utilizing ViT for image processing can result in the loss of valuable information to a certain degree.

To address this issue, we propose combining CNN with ViT. One straightforward approach is to use a hybrid model. In this hybrid model, the input image is initially processed using CNN to extract low-level features. These features are then passed to the ViT model to extract high-level features. The advantage of this approach is that it can leverage CNN's ability to preserve spatial structural information when processing image data, while also utilizing ViT's self-attention mechanism to extract higher-level features. Another approach is to employ the Vision Transformer with Convolutional Pooling (ViT-CP). In ViT-CP, we similarly use convolutional layers to preprocess the input image before passing it to the ViT model for further processing. This method reduces the computational cost of ViT. Since the convolutional layers preprocess the input data, it decreases the sequence length that the ViT model needs to handle. Additionally, this approach allows for feature extraction using ViT without sacrificing spatial structural information. The primary contributions of this paper are as follows.

- Introducing the Vision Transformer into monocular depth estimation, we incorporate a random dropout in the encoder to enhance the model's robustness and generalization performance.
- The convergence phase is divided into "coarse convergence" and "fine convergence." During the fine convergence phase, the loss is defined as the sum of segmentation loss (loss_seg), inner consistency loss (loss_in), and outer consistency loss (loss_out). This formulation quantifies the segmentation loss while considering three aspects: segmentation accuracy, internal consistency, and external consistency. By incorporating these factors into the training process, the accuracy and stability of depth estimation are further improved.
- We conducted experiments on multiple datasets and compared our approach with other methods for monocular depth estimation. The experimental results indicate significant improvements in both speed and accuracy with our method. Particularly, our approach demonstrates enhanced stability in scenarios with natural variations, showcasing its robustness.

## Method

In the context of this study, we use a self-supervised monocular depth estimation approach based on a combination of convolutional neural networks and vision converters. In this section, the method we used in detail will be described, including model structure, loss function, and training process.

### Model structure

**Figure 2:**
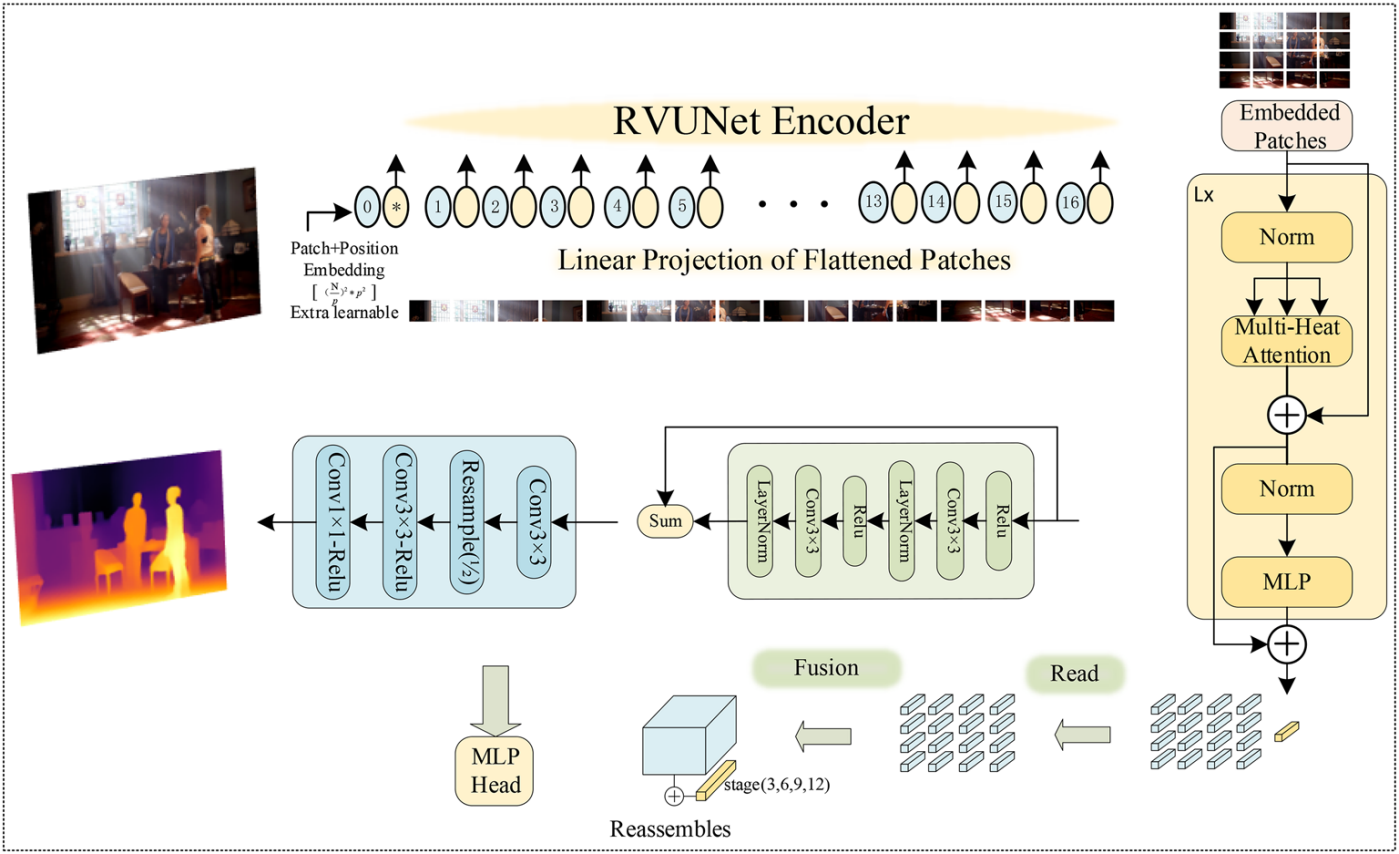
Overall architecture of AMENet.

The majority of early research predominantly employed singular convolutional modules or transformer modules for constructing network architectures. However, the latent potential of harnessing these two categories remained relatively unexplored. Thus, in our approach, we amalgamated CNN and ViT to collectively tackle the task of monocular depth estimation. Figure 2 delineates the structure of the AMENet model proposed in this study.

The input layer receives fixed-size image data. The CNN employs a sequence of convolution and pooling operations to extract image features and maps these features to a set of low-dimensional feature vectors. In this context, we utilize a pre-trained CNN model, specifically ResNet50. The ViT component involves feeding the feature vectors extracted by the CNN into the Vision Transformer. The Vision Transformer comprises a set of Transformer encoders, each consisting of multi-head self-attention mechanisms and feedforward neural networks. Through the attention mechanism, the model dynamically attends to different segments of input vectors, extracting the most information-rich feature vectors. The encoders progressively heighten the abstraction level of features, thereby generating high-dimensional representations for final classification or regression purposes. The fully connected layer concludes the architecture, mapping the features generated by the ViT to categories or regression values. This layer typically involves several hundred neurons, performing nonlinear transformations on the feature vectors to suit the requirements of various tasks.

### Loss and convergence

Due to the discrete nature of depth maps compared with their "continuous" counterparts, the loss function must account for the "uncertainty." Conversely, in the case of segmentation maps, which are also more "discrete" than "continuous", the loss function necessitates classification rather than quantification. Consequently, Mean Squared Error (MSE) loss is employed to quantify the loss for depth maps, whereas "cross-entropy" is used to classify the loss function. For given ground truth depth map and predicted depth map, the cross-entropy loss measures their similarity by quantifying the difference between them. Its formula is as follows:

1 $$ L_{depth} = - \frac{1}{N}\sum\nolimits_{i = 1}^{N} {\sum\nolimits_{j = 1}^{M} {y_{ij} \log (\hat{y}_{ij} )} } + (1 - y_{ij} )\log (1 - \hat{y}_{ij} ) $$

In the formula, $\Sigma_{i = 1}^{N}$ represents the total number of pixels in the depth map, $\Sigma_{j = 1}^{M}$ indicates the total number of depth value classes, $y_{ij}$ signifies the actual depth value at position (i,j), taking values of 0 or 1, and $\hat{y}_{ij}$ stands for the depth prediction by the model at position (i,j). In the equation, $1 - y_{ij}$ signifies the error when pixels with a depth value of 0 are predicted as 0.

In the early stages, convergence often tends to be rapid but unstable. To ensure proper convergence, it is necessary to:

- Apply a sufficiently large weight to the loss_seg term, ensuring that the predicted segmentation must be of high quality and devoid of noise;
- Apply normalized weights to loss_in and loss_out, achieved through the utilization of "scale and shift invariant loss," to ensure their proper normalization.

**Figure 3:**
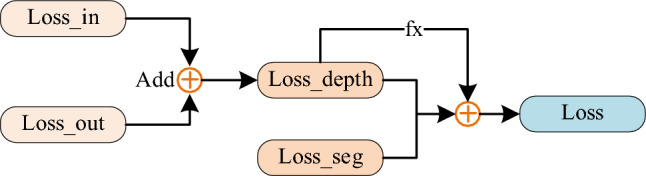
Weight correction module.

To quantify the weights among the three values, an additional correction unit is introduced, as illustrated in Fig. 3.

The magnitude of fx impacts the depth and details of the depth map. Increasing the value reduces noise, while decreasing it enhances depth details. This unit aids AMENet in producing favorable predictions even when encountering "corrupted" data.

### Encoder

At lower levels, features are both spatially accurate and of high-resolution, while at higher levels, features are spatially inaccurate yet semantically enriched. In many existing depth estimation methods^2^, ResNet is utilized as an encoder. This allows the extraction of low-resolution feature maps from high-resolution input images, capturing both semantic and spatial information correspondences. Full-dimensional dynamic convolutions^3^ address the issue of encoders' inability to model relationships between distant pixels. ACDNet^4^, on the other hand, achieves 3D reconstruction of panoramic images through an adaptive channel fusion module.

In this study, a methodology similar to ShuffleNet is employed. Feature extraction tasks are accomplished by stacking four random blocks alongside four feature extraction stages. Following each stage, the feature map's dimensions are halved, while the channel count remains consistent. The Vision Transformer is incorporated as the backbone, specifically in the encoder portion of the encoder-decoder architecture. Images with a size of N*N are divided into patches of size p*p, where each patch is sized as $(N/p)^{2}$.

For each image, segmentation is performed, followed by positional embeddings and classification embeddings operations, resulting in a matrix of size $(N/p)^{2} * 3p^{2}$, which is then fed into the ViT encoder. Additionally, to facilitate the classification task, an extra learnable special token is introduced,$x_{class} :1 * 3{\text{p}}^{2}$, as summarized by the following formula:

2 $$ {\text{z}}_{0} = [x_{class} ;x_{p}^{1} E;x_{p}^{2} E; \cdot \cdot \cdot ;x_{p}^{{\text{N}}} E;] + E_{pos} {\text{, E}} \in {\mathbb{R}}^{{(P^{2} \cdot C) \times D}} {\text{, E}}_{Pos} \in {\mathbb{R}}^{(N + 1) \times D} $$

where $x_{class}$ is the trainable label, ${\text{X}}(N,p)$ represents N patches of resolution ${\text{p}} * {\text{p}}$, *E* denotes the trainable linear projection, and ${\text{E}}_{Pos}$ signifies positional embeddings. It is important to note that the positional encoding is summed instead of concatenated. Hence, after the inclusion of positional information encoding, the input dimensions remain $(N/p)^{2} * 3p^{2} + 1) * 3p^{2}$.

In the multi-head attention module, where n denotes the number of attention heads representing the count of self-attentions and W represents the weight parameter matrix for the multi-head attention operation, which can be represented as:

3 $$ MLP(Q,{\text{K,V}}) = Concat(head_{1} , \cdot \cdot \cdot ,head_{n} )W $$

where the attention heads are defined by the following formula:

4 $$ head_{i} = Attention(QW_{i}^{Q} ,KW_{i}^{K} ,VW_{i}^{V} ) $$

$Q \in {\mathbb{R}}^{{n \times HW \times d_{k} }} ,K \in {\mathbb{R}}^{{n \times HW \times d_{k} }} ,V \in {\mathbb{R}}^{{n \times HW \times d_{k} }}$,$d_{k}$ and *d* represent the matrix multiplication and d stands for the hidden channels. In this work, we employ the Linear + Tanh activation function and introduces a dropout layer. In the experimental section, it is demonstrated that the addition of dropout enhances robustness.

Like ViT, the AMENet model is available in two variants: Base and Large, comprising 12 and 24 Transformer layers, respectively.

### Decoder

In practical applications, the purpose of monocular depth estimation is to predict distances for specific objects (such as vehicles, pedestrians, occlusions). Thus, it is of vital research significance to effectively recognize the edge texture information and localization cues of these predetermined targets. In the decoding phase, AMENet incorporates an additional class token used for classification. This is achieved by introducing a mechanism that reads out information from the token and transmits it to all other tokens:

5 $$ Read_{confusion} (t) = \{ t_{1} + t_{0} , \cdot \cdot \cdot ,t_{N} + t_{0} \} $$

**Figure 4:**
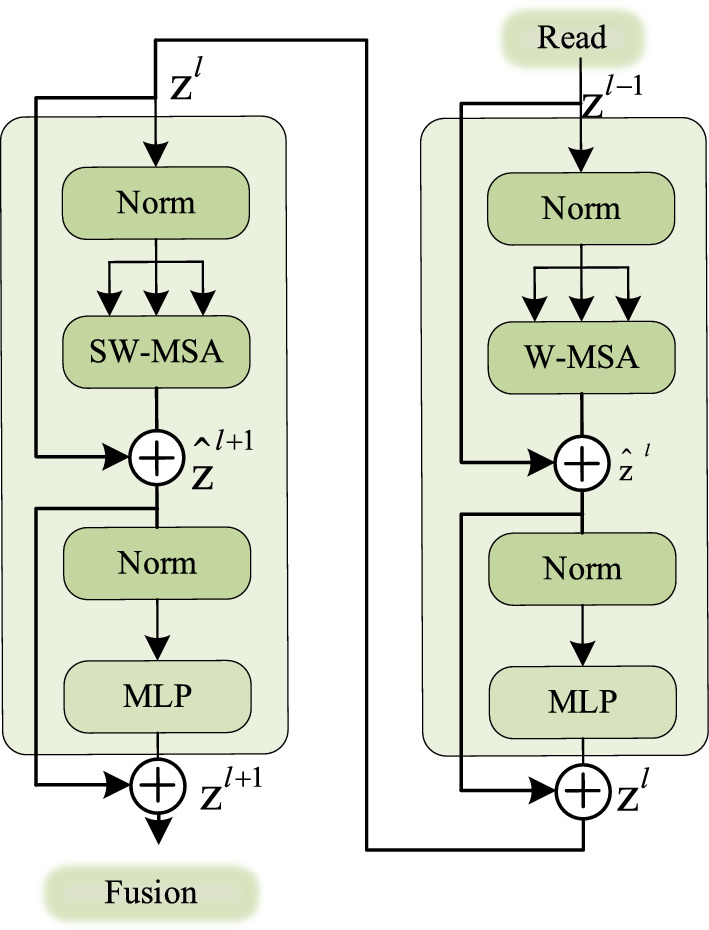
Swim windows block.

To reduce costs, as a comparative measure, we introduced the Shift Windows method from SwimTransformer during the decoding phase. Specifically, this was implemented between two consecutive Transformer Blocks, as illustrated in Fig. 4:

- The first module employs a standard Windows partition strategy, starting from the top-left corner of the feature map. An 8 × 8 feature map is segmented into 2 × 2 windows, with each window having a size of M = 4.

- The subsequent second module adopts the strategy of the moving window, where the window initiates from the position ([$\frac{M}{2},\frac{M}{2}$]) of the feature map. Subsequently, window partition operations are conducted.

As a result, there is an opportunity for interaction between different windows across two consecutive modules. Based on the moving window strategy, the computational process between two consecutive SwimTransformer Blocks is as follows:

6 $${\widehat{z}}_{l}=W-{\text{MSA}}(LN({z}_{l-1}))+{z}_{l-1},l=1\cdot \cdot \cdot L$$

7 $${z}_{l}={\text{MLP}}(LN({\widehat{z}}_{l}))+{\widehat{z}}_{l},l=1\cdot \cdot \cdot L$$

8 $${\widehat{z}}_{l+1}={\text{SW}}-{\text{MSA}}(LN({z}_{l}))+{z}_{l},l=1\cdot \cdot \cdot L$$

9 $${z}_{l+1}={\text{MLP}}(LN({\widehat{z}}_{l+1}))+{\widehat{z}}_{l+1},l=1\cdot \cdot \cdot L$$

Due to the computation of Self-Attention within local windows, each image is uniformly divided into several windows, and these windows do not overlap. Assuming each image has dimensions h*w and each window contains M*M patches, the computational complexity for MSA (Multi-Head Self-Attention) and window-based local Self-Attention is as follows:

10 $$\Omega (MSA)=4hw{C}^{2}+2(hw{)}^{2}C$$

11 $$\Omega (W-MSA)=4hw{C}^{2}+2{M}^{2}hwc$$

The time complexity has been reduced from $O({n}^{2})$ to $O(n)$.

After the reading process is completed, the generated ${\text{N}}_{p}$ is reshaped into a feature map by placing each token according to the initial position of the image. By employing spatial concatenation operations, a $\frac{H}{p} \times \frac{W}{p}$ feature map of size with D channels is generated.

12 $$ Concatenate:{\mathbb{R}}^{{N_{p} \times D}} \to {\mathbb{R}}^{{\frac{H}{p} \times \frac{W}{p} \times D}} $$

To achieve spatial downsampling and upsampling, a 1 × 1 convolution is employed to project the input to $\hat{D}$, followed by a 3 × 3 convolution. For the two models in this study, Base and Large, the operations are conducted at $l = \{ 2,5,8,11\}$ and $l = \{ 5,11,17,23\}$ layers, while $\hat{D} = 256$ represents the convolution stride and s denotes the stride.

13 $$ Resample_{s} = {\mathbb{R}}^{{\frac{H}{p} \times \frac{W}{p} \times D}} \to {\mathbb{R}}^{{\frac{H}{s} \times \frac{W}{s} \times \hat{D}}} $$

The final fusion module utilizes a residual convolution unit similar to RefineNet^5^, combining features to accomplish upsampling of the feature map.

14 $$ Reassembles_{s}^{{\hat{D}}} (t) = (Resample_{s} \otimes Concatenate)(t) $$

### Declaration of ethics

All images containing people used in this paper are from the publicly available datasets INRIA, PoseTrack, KITTI, NYU V2 and do not involve human experimentation.

### Identifiable information/image statements

All personally identifiable information/images used in this article are sourced from publicly available datasets, namely, INRIA, PoseTrack KITTI and NYU V2. The relevant statements have already been included in “Alahari, K., et al. Pose Estimation and Segmentation of People in 3D Movies. in 2013 IEEE International Conference on Computer Vision. 2013” and “Andriluka, M., et al. PoseTrack: A Benchmark for Human Pose Estimation and Tracking. in 2018 IEEE/CVF Conference on Computer Vision and Pattern Recognition. 2018”.

## Experiment

### Datadets

#### NYU Depth V2

The NYU Depth V2 dataset^6^ comprises video sequences of various indoor scenes recorded using RGB and depth camera lenses from the Microsoft Kinect device. This dataset is extensively used in depth estimation and segmentation tasks. It encompasses 464 scenes from three cities, totaling 1449 labeled RGB images and corresponding depth maps, along with 407,024 unlabeled images.

#### INRIA

The INRIA dataset^7^ consists of labeled images capturing pedestrians either running or walking. The training set comprises 614 positive samples (including 1237 pedestrians) and 1218 negative samples, while the test set contains 288 positive samples (with 589 pedestrians) and 453 negative samples. In these images, most of the human subjects are standing and are taller than 100 pixels in height. The images are primarily sourced from GRAZ-01, personal photos and Google, resulting in high clarity.

#### POSETRACK

The Posetrack dataset^8^ is derived from raw video data of the MPII dataset. It selects video segments consisting of frames 41 to 298, focusing on crowded scenes that involve multiple individuals and complex interactions between them. This selection is made with the following purpose.

- To ensure that the videos encompass a significant amount of limb movement, poses, and appearance variations.
- The dataset includes high levels of occlusions and truncations, with targets occasionally appearing partially or completely hidden and reappearing.
- Changes in human size occur within the videos due to human movement or scene scaling.
- The number of visible individuals is within the same video sequence varies.

### Evaluation metrics

The adopted evaluation metrics are as follows.

1. Absolute relative error:
  15 $$ AbsRel = \frac{1}{N}\sum\nolimits_{i = 1}^{N} {\frac{{|D_{i} - D_{i}^{*} |}}{{D_{i}^{*} }}} $$
2. Square relative error:
  16 $$ SqRel = \frac{1}{N}\sum\nolimits_{i = 1}^{N} {\frac{{|D_{i} - D_{i}^{*} |^{2} }}{{D_{i}^{*} }}} $$
3. Root mean squared error:
  17 $$ RMSE = \sqrt {\frac{1}{N}\sum\nolimits_{i = 1}^{N} {|D_{i} - D_{i}^{*} |^{2} } } $$
4. Error in logarithmic space:
  18 $$ losRMSE = \sqrt {\frac{1}{N}\sum\nolimits_{i = 1}^{N} {|\lg D_{i} - \lg D_{i}^{*} |^{2} } } $$
5. Accuracy with a threshold $T(\delta_{1} ,\delta_{2} ,\delta_{3} )$:
  19 $$ \max (\frac{{D_{i} }}{{D_{i}^{*} }},\frac{{D_{i}^{*} }}{{D_{i} }}) = \delta_{i} < T,T = \{ 1.25,1.25^{2} ,1.25^{3} \} $$

### Comparative experiments

This study's code implementation was conducted using Python 3.7 with VS Code 2019. The input image dataset was $I \in {\mathbb{R}}^{640 \times 480 \times 3}$. The training parameters were set as $epoch = 100$, utilizing the Adam optimizer, $patch\_size = 16$. When $epoch = 0$, $loss\_depth$ was set to be 0 and depth map convergence began from the segmentation map as the initial guess. Each epoch involved sampling several examples greater than or equal to 30, rather than using the entire dataset. This research was performed on Ubuntu 20.04.6 LTS, equipped with a 12th Gen Intel(R) Core(TM) i9-12900K 3.2GHz CPU and an NVIDIA GeForce RTX3090Ti 24GB graphics card, along with 2 × 32GB DDR5 memory.

In this study, a comparison was made between AMENet and several classic depth estimation networks^1,9–11^, as well as networks with improved performance in accuracy and error aspects^12–16^. Shimada et al.^13^ utilized optical flow-assisted depth estimation, DPNet^16^ leveraged pixel relationships in the spatial domain to enhance depth detail inference. AdaDepth^17^ employed adversarial learning and imposed content consistency explicitly on adapted target representations for unsupervised network training. DPT^18^ replaced convolutional networks with visual transformers as the backbone for dense prediction tasks.

The model evaluation and accuracy assessment were conducted on the KITTI dataset^19^ and the NYU Depth V2 dataset. The results indicated a certain enhancement in prediction accuracy using the proposed method. Additionally, the results were visualized to demonstrate the superiority of the proposed model.

**Figure 5:**
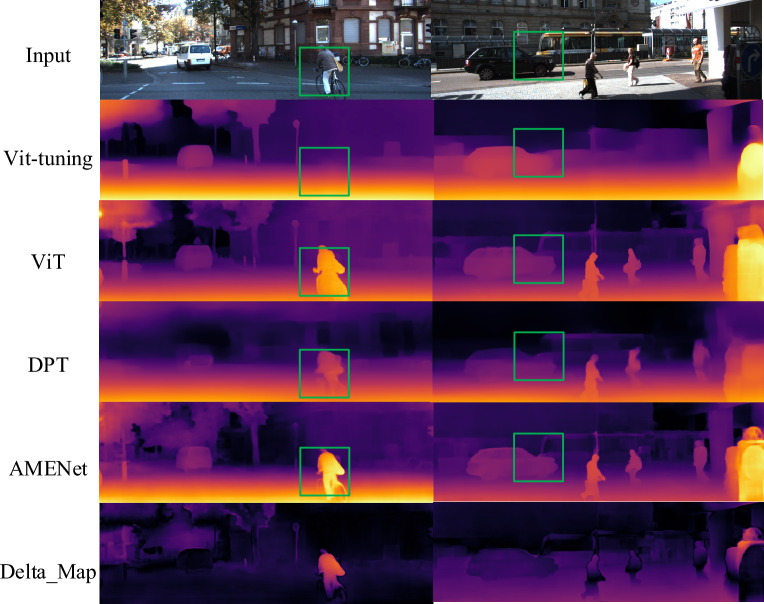
The test results on KITTI.

Figure 5 presents the experimental results of different models on the KITTI dataset. The results indicate a comparative advantage of our model over others, with clearer outlines of pedestrians in the left image and vehicle contours in the right image. The delta map illustrates the disparity between our results and the ground truth. To accentuate these differences, we have amplified the depth of the delta map from [0,50] to [0,255]. The color scale represents error magnitude, with increasing redness indicating larger discrepancies. Our model places greater emphasis on training parameters related to pedestrians, resulting in enhanced clarity but also contributing to larger errors in pedestrian-related aspects compared to other objects. Additionally, our model exhibits a less smooth handling of road distances.

As evident from Tables 1 and 2, AMENet exhibits a noticeable precision advantage, in terms of absolute relative error and root mean square error. Moreover, its accuracy aligns with the state-of-the-art models in terms of thresholds ${\updelta }_{1}$ < 1.25, ${{\updelta }_{2} < 1.25}^{2}$, ${{\updelta }_{3} < 1.25}^{3}$.

**Table 1:** Performance comparison on the KITTI Dataset.

Method	AbsRel	SqRel	RMSE	LogRMSE	$\delta_{1}$	$\delta_{2}$	$\delta_{3}$
	(Lower is better)				(Higher is better)		
Godard^20^	0.115	0.902	4.863	0.193	0.877	0.975	0.981
Kundu^21^	0.136	**0.603**	3.908	0.157	0.805	0.948	0.982
Pilzer^22^	0.144	1.007	4.66	0.24	0.793	0.923	0.968
Zhao^23^	0.308	4.995	9.614	0.437	0.684	0.795	0.897
Shu^24^	0.349	1.908	8.271	0.322	0.792	0.877	0.909
Guizilini^25^	0.112	1.082	4.124	0.165	0.867	0.927	0.968
Chen^26^	0.116	1.039	3.556	0.119	**0.879**	0.947	0.974
Bhat^27^	**0.11**	0.901	4.658	0.221	0.847	0.947	0.987
Zhang^28^	0.173	1.152	4.987	0.249	0.751	0.915	0.968
ViT^11^	0.141	1.310	6.334	0.152	0.831	0.933	0.946
Ours	0.112	1.121	4.561	**0.115**	0.851	**0.977**	**0.988**

Significant values are in bold.

**Table 2:** Performance comparison on the NYU DepthV2 dataset.

Method	REL	RMSE	$\log_{10}$	$\delta_{1}$	$\delta_{2}$	$\delta_{3}$
	(Lower is better)			(Higher is better)		
Karsch^29^	0.374	1.12	0.134	–	–	–
Li^30^	0.232	0.821	0.094	0.621	0.886	0.968
Liu^31^	0.230	0.824	0.095	0.614	0.883	0.971
Wang^32^	0.220	0.745	0.094	0.605	0.890	0.970
Eigen^1^	0.215	0.907	–	0.611	0.887	0.971
DORN^33^	0.115	0.509	0.051	0.828	0.965	0.992
Yin^34^	0.108	0.416	0.048	0.875	0.976	0.994
BTS^35^	0.110	0.392	0.047	0.885	0.978	0.994
DAV^36^	0.108	0.412	–	0.882	0.980	0.996
DPT^37^	0.110	**0.357**	**0.045**	0.904	**0.988**	0.998
ViT	0.214	0.602	–	0.762	0.851	0.902
Ours	**0.103**	0.433	–	**0.906**	0.981	**0.999**

Significant values are in bold.

**Figure 6:**
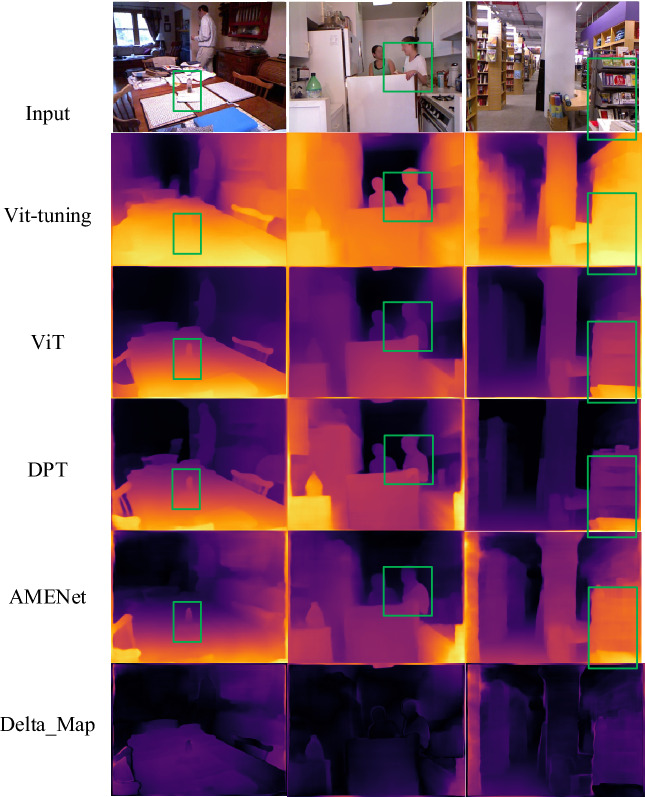
The test results on NYU V2.

Figure 6 displays the experimental results of different models on the NYU V2 dataset. The delta map reveals that our model more accurately identifies the depth information of the cup within the green box in the left image. In the middle image, our model effectively reconstructs the depth information of the person. However, for non-personal objects in the right image, the recognition of the foreground and background positions of the bookshelf and the adjacent bookshelf is not optimal.

**Figure 7:**
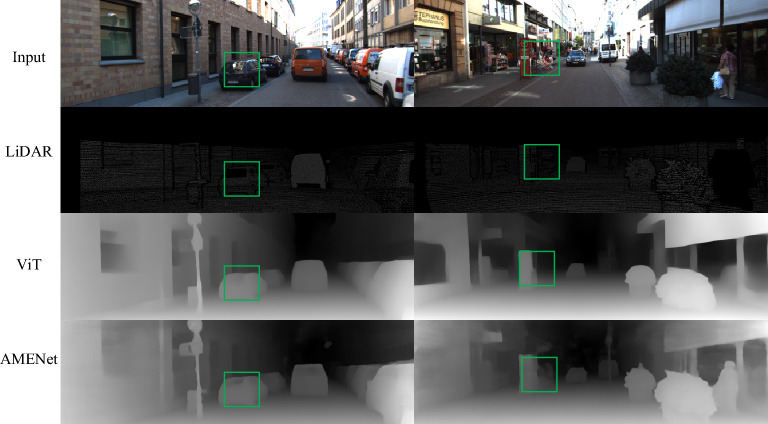
The test results on KITTI, the green boxes showcase that the proposed model handles details with minimal deviation from LiDAR measurements.

In general, the depth measurement error of LiDAR is typically small, usually at the millimeter level. The errors associated with stereo cameras are also typically within the range of a few millimeters to centimeters. Considering the depth estimation range from 5 to 80 m, the impact on model accuracy assessment is relatively minimal. We form a new validation set by combining images and depth maps captured by LiDAR and evaluate the model loss based on this dataset. The introduced discrepancy in depth values compared to LiDAR measurements is subtly elevated. As indicated in Table 3 and Fig. 7, it is evident that the proposed method remains competitive when compared to similar approaches within the same category.

**Table 3:** Performance comparison on the LiDRA form KITTI.

Method	REL	RMSE	$\log_{10}$	$\delta_{1}$	$\delta_{2}$	$\delta_{3}$
	(Lower is better)			(Higher is better)		
ViT(LiDAR)	0.219	0.615	**–**	0.747	0.834	0.844
Ours(LiDAR)	0.106	0.452	–	0.885	0.958	0.976

### Ablation study

To visually demonstrate the impact of the proposed innovations on the co-linearity of depth estimation networks, we conducted ablation experiments based on the innovations in each module. The specific results are shown in Table 3. The original network is built on the encoder network of Vision Transformer, where the encoder part consists of ResNet50, and the decoder part transforms the up-sampled output into depth values. From Table 4, it can be observed that the Weight Correction module significantly contributes to the model's accuracy, with an improvement of 0.02 in ${\delta }_{1}$ and 0.042 in ${\delta }_{3}$ . In contrast, the Window-Attention module does not show a substantial improvement in model accuracy. However, the introduction of the second attention mechanism did not result in a twofold increase in computational complexity. Instead, it allows for the same linear complexity as CNN (see Sect. 3.4 for details).

**Table 4:** Performance comparison on the KITTI dataset.

Network	Higher is better			Weight correction	Window-attention
	${\delta }_{1}$	${\delta }_{2}$	${\delta }_{3}$		
Original	0.831	0.933	0.946		
Network1	0.833	0.930	0.945		√
Network2	0.851	0.978	0.987	√	
Network3	0.851	0.977	0.988	√	√

## Conclusions

In this study, we proposed a single-monocular-depth estimation method that combines visual transformers with CNNs. We employed visual transformers as encoders to capture global receptive fields and fine-grained features. The addition of a dropout layer in the MLP and the introduction of corrective factors when handling the weights between losses contributed to enhancing the robustness of the network. Experimental results revealed that the AMENet not only minimized the loss of feature information, providing more effective information to the decoder, but also demonstrated reliable prediction performance in complex scenes and during the dealing with "corrupted" data. Although our work has demonstrated promising results, there are areas for improvement. The impact of varying sample sizes on model training at each epoch and the accuracy of added details to the depth map as the number of epochs increases require further investigation in future works.

## Data Availability

Data will be made available on request, please contact the corresponding author.
